# Predicting Additional Metastases in Axillary Lymph Node Dissection After Neoadjuvant Chemotherapy: Ratio of Positive/Total Sentinel Nodes

**DOI:** 10.3390/cancers16213638

**Published:** 2024-10-29

**Authors:** Isaac Cebrecos, Ines Torras, Helena Castillo, Claudia Pumarola, Sergi Ganau, Carla Sitges, Sergi Vidal-Sicart, Francesco Schettini, Esther Sanfeliu, Ignacio Loinaz, Marta Garcia, Gabriela Oses, Meritxell Molla, Maria Vidal, Eduard Mension

**Affiliations:** 1 Department of Obstetrics and Gynecology and Neonatology, Hospital Clinic of Barcelona, 08036 Barcelona, Spain; cebrecos@clinic.cat (I.C.); ignacio.loinaz@quironsalud.es (I.L.);; 2 Faculty of Medicine, University of Barcelona, 08007 Barcelona, Spainmolla@clinic.cat (M.M.); mjvidal@clinic.cat (M.V.); 3 Department of Radiology, Hospital Clinic of Barcelona, 08036 Barcelona, Spain; sganau@clinic.cat (S.G.);; 4 Department of Nuclear Medicine, Hospital Clinic of Barcelona, 08036 Barcelona, Spain; svidal@clinic.cat; 5 Diagnosis and Therapy in Oncology Group, August Pi i Sunyer Biomedical Research Institute (IDIBAPS), 08036 Barcelona, Spain; 6 Medical Oncology Department, Hospital Clinic of Barcelona, 08036 Barcelona, Spain; 7 Translational Genomics and Targeted Therapies in Solid Tumors Group, August Pi i Sunyer Biomedical Research Institute (IDIBAPS), 08036 Barcelona, Spain; 8 Department of Pathology, Biomedical Diagnostic Center, Hospital Clinic of Barcelona, 08036 Barcelona, Spain; 9 Department of Radiation Oncology, Hospital Clinic of Barcelona, 08036 Barcelona, Spain

**Keywords:** breast cancer, neoadjuvant chemotherapy, targeted axillary dissection, sentinel lymph node, axillary lymph node dissection

## Abstract

Background/Objectives: The aim of the study was to determine the clinical value of the sentinel lymph node ratio (SLN-R) in predicting additional positive lymph nodes during axillary lymph node dissection (ALND) in breast cancer patients following neoadjuvant chemotherapy (NAC). Methods: A cross-sectional study was performed at a single institution evaluating data from 1521 BC patients. Inclusion criteria comprised cT1/cT4, cN0/cN1 status with positive post-NAC axillary staging by SLN/TAD, respectively, and subsequent ALND. Results: The study included 118 patients, divided into two groups based on the presence or absence of additional node metastasis at ALND: 39 in the residual disease group (RD) and 79 in the non-residual disease group (nRD). Univariate logistic regression analysis of SLN-R was conducted to assess its predictive value, yielding an odds ratio (OR) of 7.79 (CI 1.92–29.5, *p* = 0.003). An SLN-R cut-off point of <0.35 was identified using ROC curve analysis, with a false-negative rate of 10.2%, as a predictor for no additional metastasis at ALND following post-NAC SLN/TAD positivity. Conclusions: The study concludes that SLN-R is a valuable predictor for determining the omission of ALND in cases where SLN/TAD is positive after NAC. This metric, in combination with other clinical variables, could help develop a nomogram to spare patients from ALND.

## Simple Summary

This study raises the question whether all breast cancer patients need axillary lymph node dissection (ALND) after neoadjuvant chemotherapy (NAC). Researchers assessed a novel clinical variable: the sentinel lymph node ratio (SLN-R) to predict additional cancerous lymph nodes during ALND. SLN-R was defined as total number of positive sentinel nodes among all sentinel nodes removed during axillary staging after NAC. Axillary surgery included Targeted Axillary Dissection (TAD) technique for cN1 breast cancer patients. We analyzed data from 1521 patients, focusing on 118 with specific cancer stages and positive sentinel /TAD nodes results after NAC. The results indicated that an SLN-R value below 0.35 could suggest a lower chance of finding more cancerous nodes, with a 10.2% false-negative rate. This means SLN-R could help identify patients who might avoid unnecessary surgeries. By combining SLN-R with other clinical factors, the study aims to create a predictive tool, enhancing personalized care and improving patients’ quality of life.

## 1. Introduction

Neoadjuvant chemotherapy (NAC) is increasingly being considered as an initial treatment option for local breast cancer (BC) patients [1], as it has demonstrated advantages such as early treatment of micrometastatic disease, the ability to reduce tumor burden, allowing more breast-conserving surgery (BCS) and the provision of tumor chemosensitivity information for future therapeutic management, especially useful in tumor subtypes like HER2+ and triple negative, for which antibody–drug conjugates and immunotherapy may be offered as additional adjuvant therapeutic management [2,3,4,5,6,7,8].

The role of NAC in managing axillary lymph nodes has significant room for improvement. NAC has the potential to reduce the staging of clinically positive axilla patients (cN1) to pathological node-negative status (ypN0) in about 40% of cases [9], which can help them avoid axillary lymph node dissection (ALND) and its associated complications, such as lymphedema, pain and reduced arm mobility [10]. However, many patients still undergo ALND because SLN remains positive after NAC (ypN+) when surgically staging the axilla.

In light of the findings from the AMAROS and ACOSOG-Z11 trials in upfront surgery [11,12], allowing up to two macrometastases in SLN, ongoing trials in the neoadjuvant setting are focused on identifying which patients benefit the most from ALND and if any nodal residual burden disease could be allowed without compromising oncological outcomes [13].

For clinically negative axilla (cN0) BC patients, sentinel lymph node biopsy (SLN) after NAC was proven to be feasible and safe according to NSABP-B32 trial results [14]. However, in cN1 BC patients, the SLN could be impaired by NAC-induced fibrosis in lymphatic drainage and nodes [15], leading to unacceptable false-negative (FN) rates if SLN was performed alone [16,17,18]. For this reason, targeted axillary dissection (TAD), which involves marking the positive node before chemotherapy and providing its selective identification and removal within SLN, has become (or is becoming) a worldwide extended method for staging cN1 patients after NAC [19,20]. Moreover, TAD allows ALND to be avoided in those cN1 patients who present ypN0 after NAC. However, most post-NAC patients who present any positivity after SLN or TAD (ypN+) are still undergoing ALND.

The benefit of ALND in patients presenting ypN+ after NAC is currently being investigated. The NSABP B-27 trial reported 56% of patients without further positive non-sentinel nodes (non-SLN) when completing ALND due to positive SLN post-NAC [21]. Moreover, our group recently reported only 22% of additional positive non-SLNs in a selected cN0 group of patients with positive SLN (ypN+) after NAC [22].

Thus, there is a need to evaluate clinical factors associated with ALDN residual disease in the post-NAC scheme that might allow physicians better select patients not suitable to complete ALDN in this scenario.

In the line of existing tools for predicting further positive lymph nodes after SLN in upfront surgery scenario [23], the aim of this observational cross-sectional study was to assess the power of the ratio of positive SLN-TAD/total SLN-TAD nodes removed during axillary staging after NAC (SNL-R) to predict additional residual disease at ALND. Secondary objectives of the study were to assess correlation of epidemiological and clinical variables to ALND residual disease.

## 2. Methods

### 2.1. Study Design

This is a cross-sectional study performed through a prospectively maintained surgical database including all BC patients treated at the Hospital Clinic of Barcelona (HCB) from January 2016 to July 2022. This study followed the Strengthening the Reporting of Observational Studies in Epidemiology (STROBE, https://www.equator-network.org/reporting-guidelines/strobe/, accessed on 14 September 2024) reporting guidelines [24].

### 2.2. Patient Selection

The study was approved by the local ethical committee (HCB/2022/0791). Informed consent was requested from all patients. Data collection followed the principles outlined in the Declaration of Helsinki.

Data were extracted from all infiltrating BC patients at clinical cT1-cT4c and cN0-cN1 stages who received NAC as primary systemic treatment and in the posterior axillary surgery underwent ALND (Berg levels I and II ± III) as a result of positive SLN/TAD at the time of surgery (ypN+).

**Figure 1:**
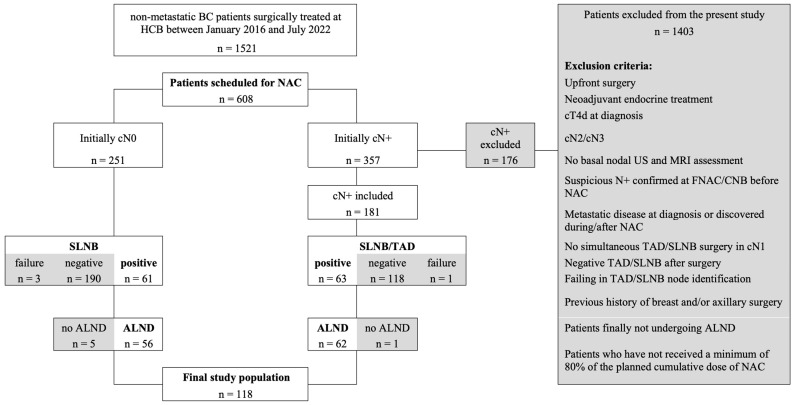
Flowchart of patients according to initial cN status undergoing NAC. Bold boxes refer to excluded patients. List of abbreviations: BC—breast cancer. HCB—Hospital Clinic of Barcelona. NAC—neoadjuvant chemotherapy. *c*—clinical. SLN—sentinel lymph node biopsy. TAD—targeted axillary dissection. ALND—axillary lymph node dissection. US—ultrasound. MRI—magnetic resonance imaging. FNAC—fine-needle aspiration cytology. CNB—core needle biopsy.

Patients with a result of ypN0 in axillary staging, inflammatory BC (cT4d) or cN2/3 BC patients at diagnosis or during/after NAC were excluded from this study (Figure 1).

### 2.3. Diagnosis and Pathologic Evaluation

Infiltrating BC was diagnosed in all patients using core needle biopsy (CNB) after breast mammography and/or ultrasound (US) examination performed as a result of either screening programs or breast referring symptoms. Specimen evaluation included estrogen and progesterone status (ER, PR), HER2 overexpression and Ki67 assessment determined by immunohistochemistry (IHC) [25,26]. In case of moderate overexpression of human epidermal growth factor receptor 2 (HER2), in situ hybridization was used to determine gene amplification [27].

Initial axillary status was determined in all patients by both clinical examination and axillary imaging with ultrasound (US) and magnetic resonance imaging (MRI), which was further investigated with fine-needle aspiration biopsy (FNAB) in case of suspicious lymph nodes. All cN1 patients included in the study had a US-guided FNAB confirmed positive. Axillary lymph nodes matted at diagnosis (cN2) were excluded from the study; as per institution guidelines, all cN2 patients were candidates to ALDN after completing NAC. Breast and axillar response to NAC was assessed by MRI using RECIST criteria within 1–3 weeks after the last dose of chemotherapy [28]. In cN1 biopsy proven cases, a clip was placed in the involved node just before starting NAC in order to perform TAD surgery afterwards [29]. All cN1 clipped patients were included in the study independently of clinical and radiological response after NAC. As per institution protocol, if more than one node was diagnosed in cN1 patients, only the larger node was clipped and removed during TAD.

### 2.4. Management and Treatments

After a Tumor Board discussion, patients received NAC ± anti-HER2 according to international guidelines [1,30,31]. Most chemotherapy regimens included anthracyclines (Epirubicin or Adriamicin), in combination with sequential Taxanes (Docetaxel or Paclitaxel). Trastuzumab +/− Pertuzumab was given to all HER2-positive patients.

Breast surgery included breast-conserving surgery or mastectomy with or without immediate reconstruction; axillary surgery consisted of SNL vs. TAD depending on initial nodal status (cN0/cN1, respectively). To identify SLN, preoperative lymphoscintigraphy with using intratumoral/peritumoral injection of ^99^Tc labeled radiocolloid was performed [32,33]. Simultaneously, under ultrasound guidance, an iodine-125 radioactive seed was placed in lymph nodes of cN1 patients who had been previously marked, according to TAD technique described by Caudle et al. [29,34]. No dual tracer was used during SLN/TAD procedures as per institutional protocol. There was not a minimum fixed number of lymph nodes deemed acceptable to retrieve.

SLNs and clipped nodes were singled out with a gamma-probe and analyzed intraoperatively. Histopathological examination was performed using the standard method (frozen section and further hematoxylin/eosin + immunohistochemistry) or the one-step nucleic acid amplification (OSNA) technique according to CK19 expression on CNB, obtaining a result of macrometastases (≥2 mm), micrometastases (≥0.2 to 2 mm) or isolated tumor cells (≤0.2 mm) [35]/macrometastasis (OSNA ≥ 5 × 10^3^ copies/μL of CK19 mRNA), micrometastasis (2.5 × 10^2^–5 × 10^3^ copies/μL), isolated tumor cells (1.6 × 10^2^–2.5 × 10^2^ copies/μL) and non-metastasis (<1.6 × 10^2^ copies/μL) [36]. Completion of ALDN was performed up to the second axillary level in all included patients that presented with a positive SLN/TAD. The third axillary level was only included if palpable nodes were detected during surgery. Adjuvant systemic therapy and radiotherapy were offered according to treatment response. And adjuvant radiotherapy was administrated according to the clinical and pathological stage as indicated by the international guidelines [37].

### 2.5. Variables of Study

The main descriptive outcome assessed in the study was the identification of residual disease in the completed ALND, which served to separate two groups of study to evaluate outcomes’ association to additional metastasis in ALND: residual disease in ALND (RD-group) and non-residual disease in ALND (nRD-group).

Descriptive analysis of epidemiologic, diagnostic and molecular data of the BC was performed. Surgical details such as the total number of SLN/clipped nodes and ALND total nodes and total number of SLN/clipped node and ALND metastasis were described.

### 2.6. Definition of Sentinel Lymph Node Ratio (SLN-R)

As the main outcome, SLN-R was defined as total number of positive SLN and/or clipped nodes among the total number of SLN and/or clipped nodes removed during SLN/TAD procedures, independently of lymph node metastasis size (the clipped node in TAD was accounted jointly with SLN for statistical analysis).

### 2.7. Statistical Analysis

Statistical analyses were performed with the STATA software, version 15.1 (StataCorp LLC, College Station, TX, USA). Continuous and normally distributed variables were presented as mean ± standard deviation. Categorical variables were presented as absolute values and percentages. Univariate comparisons were performed using Student’s *t* test, Pearson’s Chi-square test or Fisher’s exact test. Statistical significance was defined as a *p* value < 0.05. An ROC curve analysis and an optimal cut-off point for maximum efficiency were calculated [38]. Logistic regression was used to estimate odds ratios (ORs) and their 95% confidence intervals (95% CIs) for the association of SLN-R with axillary status after ALND. Missing data were handled using pairwise deletion.

## 3. Results

### 3.1. Study Population Characteristics

A total of 1521 cN0-cN1 BC patients were identified to be included in the study. Of them, 608 (40%) were scheduled for NAC after a Tumor Broad Committee discussion. Post-NAC axillary SLN resulted positive in 61 (24.3%) cN0 patients, while combined SLN/TAD procedures were positive in 63 (34.8%) cN1 patients. ALND was completed in 118/124 of these ypN+ patients (95.2%), which were included in the present study. Of these 118 patients, 58.5% were postmenopausal, and the basal tumor dimension based on magnetic resonance imaging (MRI) was 34 mm (SD ± 19.6 mm). Identification rates for SLN and TAD procedures were 98.8% and 99.4%, respectively. Study population characteristics are resumed in Table 1.

During axillary surgery, the OSNA technique was performed for node assessment in 76 (64.4%) patients, while conventional intraoperatively frozen section was used in the remaining 42 (35.6%) patients. Among cN1 patients scheduled for SLN/TAD procedures after NAC, coincidence between iodine-125 seed clipped node and 99m-TC hot node was observed in 50 (80.6%) patients. All patients within this study had a positive axillary staging surgery after NAC, either by SLN and/or TAD, and the mean number of nodes removed was 3.03 (SD ± 1.53), resulting in three or more nodes being removed in 60.2% of cases. The mean number of positive nodes was 1.55 (SD ± 0.76), with macrometastasis observed in 61% of cases.

During ALND, the mean number of nodes removed was 12 (SD ± 6.2). In this population, in-breast pathologic complete response (PCR), meaning absence of infiltrating BC in the surgical specimen, was achieved in 13 (11%) cases. Axillary surgery results are summarized in Table 2.

### 3.2. Association Between Clinical–Pathological Factors and Residual Disease in ALND

Two groups were evaluated: the RD-group (n = 39; 33.1%) and the nRD-group (n = 79; 66.9%). There were no differences between the two groups in relation to the epidemiologic and clinical characteristics such as age, BMI, menopause status, histology type, tumor grade, tumor focality, combination of NAC treatments and type of breast surgery.

However, in the univariate logistic regression analysis, higher tumor size by MRI (continuous, *p* = 0.004), higher cT and cN stage at diagnosis (*p* = 0.021 and *p* < 0.005, respectively) and breast MRI response after NAC (non-complete vs. complete, *p* = 0.03) were significantly associated with presenting residual disease at ALND.

On the other hand, higher ER and PR levels (*p* = 0.034 and *p* = 0.017, respectively), Ki67 value (*p* = 0.029) and IHQ type (luminal vs. TN and HER2+, *p* = 0.047) and as well SLN positivity burden (macrometastasis vs. ITC/micromestastasis, *p* < 0.001) were also associated with presenting residual disease at ALND. Summarized results for univariate analysis are shown in Table 3.

### 3.3. Analysis for SLN-R

Finally, a specific analysis of the SLN-R was performed. In the RD-group, the mean SLN-R was 0.73, compared to 0.56 in the nRD-group. Univariate logistic regression analysis of SLN-R was performed, showing significant association between higher SLN-R and RD (OR = 7.79, 95% CI: 1.92–29.5, *p* = 0.003).

ROC curve analysis of the optimal cut-off point to achieve minimum FN rate resulted in a cut-off point of 0.35, OR: 5.08, *p* = 0.001. Using the obtained cut-off, 29/118 (24.6%) patients could have been spared from ALND with an FN rate of 10.2%, a sensitivity of 89.7%, a specificity of 36.7%, a positive predictive value of 41.2% and negative predictive value of 87.9%. However, of those classified as SLN-R > 0.35, 50/85 (58%) would not have presented additional residual disease at the ALND.

## 4. Discussion

In this study, the SLN-R was assessed to be a potential predictive tool for residual disease in ALND in BC patients presenting positive SLN/TAD after NAC. Lower values for SLN-R (SLN-R ≤ 0.35) were shown to be independently associated in univariate analysis to not presenting further lymph node involvement when performing ALND, and this cut-off was proven to have a 10.2% FN rate when tested in an ROC curve. For this reason, the use of this straightforward SLN rate may be one of the pillars to the present de-escalation of ALND in the post-NAC axillary surgery scheme, with potential to avoid around 25% of the non-RD currently still undergoing ALND and thus spare this group of patients from potential severe secondary effects such as lymphedema.

Nowadays, any nodal residual deposit after NAC in SLN represents tumor cell clones resistant to treatment, and it remains an indication for ALND in BC main patients’ guidelines [39,40,41], which entails higher risk for arm lymphedema and shoulder mobility impairment [42]. However, residual disease in ALDN in BC patients with positive SLN after NAC ranges from 36.6 to 77.8%; thus, a significant portion of the population may be receiving overtreatment [43,44,45,46].

SLN-R has been explored in primary surgery for predicting additional non-SLN metastasis [47], but in light of recently SENOMAC trial results, its clinical applicability in the upfront surgery context is scarce [48]. On the other hand, other axillary node ratios (number of positive lymph nodes, including SLN and ALND, out of the total number of lymph nodes) have been studied as a predictor of disease-free survival (DFS) and overall survival (OS) of patients undergoing ALND after NAC and have been proposed as a new alternative ypN staging method [49,50], but there are few works exploring SLN-R value for predicting non-SLN involvement in the neoadjuvant setting. Leonardi et al. [45] reported a higher ratio of positive SLNs/total SLNs (*p* = 0.016) as well as patients’ age, cN+ status before NAC and nodal extracapsular extension to be predictive for non-SLN metastasis at multivariate analysis for any tumor burden in SLN after NAC, in a consecutive series of 265 patients from a single institution. However, in this study, cut-offs for age and nodal ratio were arbitrarily chosen, considering 50 years as the limit for postmenopausal status and a nodal ratio of ≤0.5 as the maximum value, whereas in our study, we restricted the SLN-R value to ≤0.35, in order minimize the FN rate. Moreover, in contrast with Leonardi et al., the present study reports a very high rate of three or more SLNs removed at SLN/TAD performance (60.2% vs. 32.1%), which supplies very good lymph node representation at axillary staging after NAC.

In another recent study, prediction of RD in ALND in BC patients with positive SLN after NAC was attempted, using a nodal sentinel ratio ≥ 0.5 (OR = 6.5, 95% CI: 41.7–23.7), with cN+ status at diagnosis (OR = 18.3, 95% CI: 4.0–83.6) proving to be an independent risk factor for residual axillary disease. The sensitivity and negative predictive value of a ratio of positive nodes in SLN ≥ 0.5 were 87% (95% CI: 75.1–94.6%) and 75% (95% CI: 55.1–89.3%), respectively [51].

**Figure 2:**
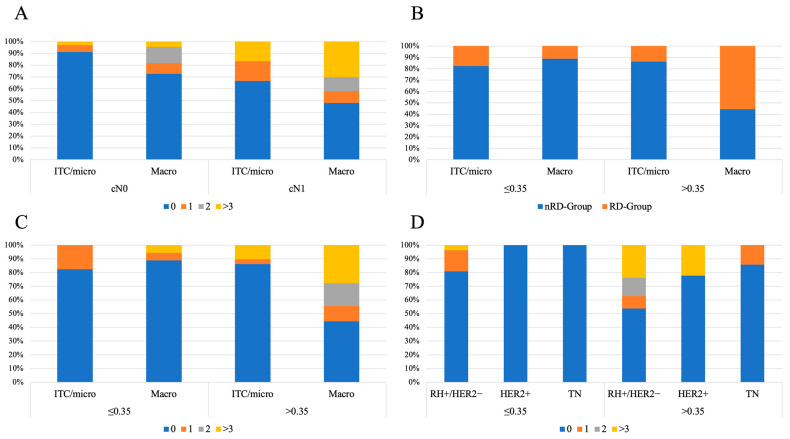
Patterns of SLN, SLN-R and non-SLN involvement at ALND. (**A**) Number of non-SLN affected at ALND, according to cN status at diagnosis and size of SLN metastasis in the overall population. (**B**) Pattern of non-SLN involvement after ALND, according to SLN-R cut-off ≤ 0.35. (**C**) Number of non-SLN affected at ALND, according to SLN-R cut-off ≤ 0.35 and size of SLN metastasis. (**D**) Number of non-SLN affected at ALND, according to SLN-R cut-off ≤ 0.35 within each IHC subtype. List of abbreviations: nRD—non-residual disease; RD—residual disease; IHC—immunohistochemistry; SLN—sentinel lymph node; ALND—axillary lymph node dissection; ITC—isolated tumor cells; Micro—micrometastases; Macro—macrometastases; HR—hormone receptor; + positive; − negative; TN—triple negative breast cancer. *p* values refer to Chi-squared tests.

Regarding molecular subtypes, tumor biology is one of the strongest parameters to predict positive axillary lymph nodes after NAC [52], and in the present study, it was observed that the probability of RD in ALND was lower for HER2-enriched and TN tumors in comparation with HR+ tumors for SLN-R ≤ 0.35 values (Figure 2D). Since HER2-like and TN tumors’ chemosensitivity leads to higher pCR rate [53], this population is less represented in this study, and the association of SLN-R < 0.35 for non-RD in ALND when SLN meets positivity must be considered with caution.

Besides SLN positivity, initial breast tumor burden and residual in-breast tumor response after NAC may provide valuable information to be taken into account for non-RD prediction. In this study, higher ypT status was associated (*p* = 0.008) with the RD-group. Furthermore, low SLN tumor burden (ITC/micrometastasis) was associated to non-RD (*p* = 0.001), while a higher SLN-R and larger metastasis size were associated with more extensive and persistent disease at ALND (Figure 2B,C). Some authors suggested that SLN micrometastasis may predict RD in up to 63% of ALND [44], but according to previous findings, SLN size metastasis may have different prognostic implications depending on whether patients were initially classified as cN0 or cN+ [22] (Figure 2A).

To the best of our knowledge, this is the first study including cN1 patients submitted to TAD after NAC using SLN-R as a new clinical feature for predicting non-SLN involvement. Therefore, in the presence of SLN metastasis after NAC (ypN+), we should consider whether undergoing an ALND is really providing any benefit to our patients. We should not focus only on which axillary surgery is performed after NAC (SLNB, TAD or ALND) but also on improving oncologic outcomes as a final goal for our patients, in light of recent studies showing no survival benefit of ALND over SLNB after NAC in an exclusively node positive luminal-like BC population [54]. Other works have also questioned TAD vs. SLNB in the neoadjuvant context, showing that single-tracer SLNB achieves similar oncological outcomes to TAD [55]. To date, current guidelines still lead physicians to overtreat many patients, so the scientific community’s responsibility is to provide tools to better select patients who can be safely spared from ALND. SNL-R, despite not being ideal, is the first safe tool to start sparing unnecessary ALND to up to 25% of patients still receiving an axillary overtreatment.

Nonetheless, this study presents some limitations:

First, extracapsular nodal involvement was not uniformly reported and could not be included as a variable in our analysis because the one-step nucleic acid (OSNA) technique was performed to assess SLN intraoperatively in most cases (64.4%) [56,57].

Second, due to being focused on patients presenting ypN+ after NAC, the cohort of analysis is selecting BC tumors with low probability of PCR after NAC, observing most of the cases presenting HR+. However, these patients are the most potential beneficiaries of a downstaging of ALND, since other molecular subtypes such as HER2 positive and triple negative very frequently present PCR in axilla and thus are already not suitable for ALND. Furthermore, we are not providing SLN-R results by tumor subtype, and it should be emphasized that higher TN/HER2 positive BC tumors’ chemosensitivity may have led to a selection bias, making it difficult to generalize the results as these populations are underrepresented.

In addition, in this study, only 39 patients (33.1%) presented RD at ALND. This represents a lower rate of further nodal affection compared to data from other series (range from 22.2% to 63.4%) [22,43,44,45,46]. This low rate could be explained by initial nodal status assessment by ultrasound and a high rate of HR+ patients (78.8%) included in the study. While previous studies do not refer to the diagnostic method of lymph node status (cN0/cN+), in this study, routine use of axillary ultrasound may lead to bias, detecting a higher rate of cN1 patients with minimal axillary involvement before NAC.

Finally, the future of axillary surgery will be shaped by ongoing trials like the ATNEC trial [58], which compares nodal radiotherapy to axillary nodal clearance in breast cancer patients achieving ypN0 status after NAC, aiming to enhance disease-free survival and reduce lymphedema.

## 5. Conclusions

This study highlights SLN-R as a new clinical variable to be used when deciding whether to perform ALND after NAC in cases of positive SLN. SLN-R ≤ 0.35 alone presents an acceptable 10.2% FN rate and provides valuable axillary staging information for considering omitting ALND. Moreover, SLN-R might be combined with other clinical variables and could form the basis of a predictive future nomogram of ALND residual disease in the neoadjuvant setting.

## Figures and Tables

**Table 1:** Overall population demographics and distribution according to ALND results. Significant *p* values are reported in bold. ^a^ Only 2 cT4b patients fulfilled the inclusion criteria in the cT3/cT4 category; the remainder of the group is composed exclusively of cT3 patients. ^b^ For statistical analysis, RECIST criteria categories of progressive disease, stable disease and partial response have been aggregated into a non-complete response group. ^c^ Axillary US and breast/axillar MRI were performed at initial diagnosis in all patients included in the study, and breast/axillar MRI after NAC was also scheduled in all patients, except in 4 patients whose data are not available. List of abbreviations: ALND—axillary lymph node dissection; SLN—sentinel lymph node; SD—standard deviation; BMI—body mass index; MRI—magnetic resonance imaging; ER—estrogen receptor; PR—progesterone receptor; IHC—immunohistochemical; HR—hormonal receptor; TN—triple negative; NAC—neoadjuvant chemotherapy.

	Overall Population		Non-RD Negative Non-SLN at ALND Group		RD Positive Non-SLN at ALND Group		*p* Value
	N	%	N	%	N	%	
	118	100.0	79	66.9	39	33.1	
*Age at diagnosis (years)*							
Mean	54.9	−	55.0	−	54.8	−	
SD	±13.0	−	±13.4	−	±12.4	−	0.934
*BMI* (Kg/m^2^)							
Mean	25.1	−	24.5	−	26.4	−	
SD	±4.9	−	±4.1	−	±6.2	−	0.056
*Menopause at diagnosis*							
Yes	69	58.5	48	60.8	21	58.5	
No	49	41.5	31	39.2	18	41.5	0.553
*Tumor size by MRI* (mm)							
Mean	34	−	30.4	−	41.4	−	
SD	±19.6	−	±15.1	−	±25.2	−	**0.004**
*cT stage*							
cT1	27	22.9	23	29.1	4	10.3	
cT2	70	59.3	46	58.2	24	59.3	
cT3/cT4 ^a^	21	17.8	10	12.7	11	17.8	**0.021**
*cN stage*							
cN0	56	47.5	47	59.5	9	47.5	
cN1	62	52.5	32	40.5	30	52.5	***p* < 0.005**
*Histology type*							
Ductal	99	83.9	68	86.1	31	79.5	
Lobular	13	11.0	6	7.6	7	18.0	
Other	6	5.1	5	6.3	1	2.5	0.195
*Tumor grade*							
I	19	16.1	13	16.5	6	15.4	
II	80	67.8	49	62.0	31	79.5	
III	19	16.1	17	21.5	2	5.1	0.054
*ER%*							
Mean	77	−	72.6	−	85.9	−	
SD	±32.2	−	±36.7	−	±17.6	−	**0.034**
*PR%*							
Mean	47.9	−	42.0	−	59.9	−	
SD	±38.7	−	±38.1	−	±36.3	−	**0.017**
≥20%	42	35.6	34	81.0	8	19.0	
<20%	76	64.4	45	59.2	31	40.8	**0.024**
*Ki67*							
Mean	29.5	−	32.1	−	24.5	−	
SD	±17.6	−	±18.9	−	±13.5	−	**0.029**
≤14%	17	14.4	9	52.9	8	47.1	
>14%	95	80.5	65	68.4	30	31.6	0.268
*IHC tumor classification*							
HR+/HER2 negative	93	78.8	57	72.2	36	92.3	
HER2 positive	15	12.7	13	16.5	2	5.1	
TN	10	8.5	9	11.4	1	2.5	**0.047**
*Tumor focality*							
Unifocal	72	61.0	46	58.2	26	66.7	
Multifocal/multicentric	46	39.0	33	41.8	13	33.3	0.426
*NAC treatment*							
Anthracyclines + taxanes	90	76.3	58	73.4	32	82.1	
Taxanes	9	7.6	5	6.3	4	10.3	
Anti-HER2 agents	15	12.7	13	16.5	2	5.1	
Others	4	3.4	3	3.8	1	2.6	0.306
*Breast MRI response after NAC*							
Complete response	26	22.0	22	27.9	4	10.3	
Non-complete response ^b^	88	74.6	53	67.1	35	89.8	
Not available ^c^	4	3.4	4	5.1	0	0.0	**0.030**
*Breast surgery*							
Conservative	61	51.7	40	50.6	21	53.9	
Mastectomy	57	48.3	39	49.4	18	46.2	0.845

**Table 2:** Axillary surgery details according to ALDN results. Significant *p* values are reported in bold.

	Overall Population		Non-RD Negative Non-SLN at ALND Group		RD Positive Non-SLN at ALND Group		*p* Value
	N	%	N	%	N	%	
	118	100.0	79	66.9	39	33.1	
*Number of SLN/TAD removed*							
Mean	3.03	−	3.09	−	2.92	−	
SD	±1.53	−	±1.55	−	±1.49	−	
Range	1–7	−	1–7	−	1–7	−	0.5774
1	22	18.6	14	17.7	8	20.5	
2	25	21.2	17	21.5	8	20.5	
≥3	71	60.2	48	60.8	23	59.0	0.926
*Number of positive SLN/TAD*							
Mean	1.55	−	1.38	−	1.90	−	
SD	±0.76	−	±0.61	−	±0.91	−	
Range	1–4	−	1–3	−	1–4	−	***p* < 0.001**
1	70	59.3	54	68.4	16	41.0	
2	33	28.0	20	25.3	13	33.3	
≥3	15	12.7	5	6.3	10	25.6	**0.004**
*Type of positive SLN/TAD*							
ITC/micrometastasis	46	39.0	39	49.4	7	17.9	
Macrometastasis	72	61.0	40	50.6	32	82.1	**0.001**
*SLNRatio 1/2*							
0.00–0.50	62	52.5	49	62.0	13	33.3	
0.51–1.00	56	47.5	30	38.0	26	66.7	**0.006**
*SLNRatio 1/3*							
0.00–0.33	35	29.7	30	38.0	5	12.8	
0.34–0.66	30	25.4	20	25.3	10	25.6	
0.67–1.00	53	44.9	29	36.7	24	61.5	**0.008**
*Nodes removed at ALND*							
Mean	12.0	−	11.1	−	13.8	−	
SD	±6.2	−	±5.0	−	±7.9	−	
Range	1–35	−	1–25	−	2–35	−	**0.03**
≤9	43.0	36.4	30	38.0	13	33.3	
>9	75.0	63.6	49	62.0	26	66.7	0.69
**Residual pathological features after NAC**							
*Tumor size* (mm)							
Mean	16.9	−	14.03	−	22.7	−	
SD	±15.7	−	±14.13	−	±17.2	−	
Range	0–80	−	0–61	−	2–80	−	**0.0043**
*ypT*							
ypT0/ypTis	13	11.0	13	16.5	0	0.0	
ypT1	68	57.6	47	59.5	21	53.8	
ypT2	31	26.3	16	20.3	15	38.5	
ypT3	6	5.1	3	3.8	3	7.7	**0.008**
*ypN*							
ypN0 (mol+)	8	6.8	8	10.1	0	0.0	
ypN1mi	33	28.0	31	39.2	2	5.1	
ypN1	50	42.4	40	50.6	10	25.6	
ypN2	22	18.6	0	0.0	22	56.3	
ypN3	5	4.2	0	0.0	5	12.8	***p* < 0.001**

List of abbreviations: SLN—sentinel lymph node; ALND—axillary lymph node dissection; TAD—targeted axillary dissection; SD—standard deviation; ITC—isolated tumor cells; NAC—neoadjuvant chemotherapy.

**Table 3:** Univariate analysis. Prediction clinical features of additional residual disease at ALND.

	OR	SE	CI	*p* Value
*Primary tumor size*	1.03	0.01	1.008–1.051	0.005
*Clinical nodal status at diagnosis (cN1* vs. *cN0)*	4.90	2.17	2.05–11.68	<0.001
*IHC tumor classification*				
HER2 positive	1	−	−	
TN	0.72	0.94	0.057–9.217	
HR+/HER2 negative	4.11	3.24	0.874–19.266	0.02
*Radiological MRI Complete response (yes* vs. *no)*	0.28	0.16	0.087–0.867	0.01
*Number of positive SLN and/or TAD*				
1	1	−	−	
2	2.19	1.00	0.897–5.362	
≥3	6.75	4.17	2.013–22.632	0.004
*Type of positive SLN/TAD metastasis*				
ITC/micrometastasis	1	−	−	
Macrometastasis	4.46	2.11	1.760–11.287	<0.001

List of abbreviations: OR—odds ratio; SE—standard error; CI—confidence interval; IHC—immunohistochemical; TN—triple negative; HR—hormonal receptor; MRI—magnetic resonance imaging; SLN—sentinel lymph node; TAD—targeted axillary dissection; ITC—isolated tumor cells.

## Data Availability

All data were collected in an electronic database and managed in accordance with privacy regulations. Cebrecos I. and Mension E. had full access to all the data in the study and take responsibility for the integrity of the data and the accuracy of the data analysis. Data and materials will be shared when requested on individual demand to the corresponding author.
